# A randomized trial of specialist genetic assessment: psychological impact on women at different levels of familial breast cancer risk

**DOI:** 10.1038/sj.bjc.6600051

**Published:** 2002-01-21

**Authors:** K Brain, P Norman, J Gray, C Rogers, R Mansel, P Harper

**Affiliations:** Institute of Medical Genetics, University of Wales College of Medicine, Heath Park, Cardiff CF14 4XN, UK; Department of Psychology, Sheffield University, Western Bank, Sheffield S10 2TP, UK; Institute of Medical Genetics, University Hospital of Wales NHS Healthcare Trust, Heath Park, Cardiff CF14 4XN, UK; Breast Test Wales, 18 Cathedral Road, Cardiff CF1 9LJ, UK; Department of Surgery, University of Wales College of Medicine, Heath Park, Cardiff CF14 4XN, UK

**Keywords:** familial breast cancer, genetic risk assessment, psychological impact, service delivery

## Abstract

The aim was to compare the psychological impact of a multidisciplinary specialist genetics service with surgical provision in women at high risk and those at lower risk of familial breast cancer. Women (*n*=735) were randomized to a surgical consultation with (trial group) or without (control group) specialist genetic risk assessment and the possible offer of presymptomatic genetic testing. Participants completed questionnaires before and immediately after the consultation to assess anxiety, cancer worry, perceived risk, interest in genetic testing and satisfaction. Responses of subgroups of women stratified by clinicians as low, moderate, or high risk were analyzed. There were no significant main effects of study intervention on any outcome variable. Regardless of risk information, there was a statistically significant reduction in state anxiety (*P*<0.001). Reductions in cancer worry and perceived risk were significant for women at low or moderate risk (*P*<0.001) but not those at high risk, and satisfaction was significantly lower in the high risk group (*P*<0.001). In high risk women who received specialist genetic input, there was a marginally significant trend towards increased perceived risk. The effect of risk information on interest in genetic testing was not significant. Breast care specialists other than geneticists might provide assessments of breast cancer risk, reassuring women at reduced risk and targeting those at high risk for specialist genetic counselling and testing services. These findings are discussed in relation to the existing UK Calman-Hine model of service delivery in cancer genetics.

*British Journal of Cancer* (2002) **86**, 233–238. DOI: 10.1038/sj/bjc/6600051 www.bjcancer.com

© 2002 The Cancer Research Campaign

## 

Breast cancer is the most common cancer in women in the UK. Advances in knowledge of the genetic basis of breast cancer have led to increased public awareness of the importance of a family history in determining susceptibility to the disease. However, the vast majority of breast cancer cases arise sporadically, with no clear familial genetic component. Known inherited gene mutations are implicated in around 5–10% of cases (King *et al*, 1993), therefore presymptomatic genetic testing is not appropriate for the majority of women with a family history of breast cancer. In recent years there has been an increase in referrals to specialist cancer genetics services which offer genetic risk assessment and counselling and, in the small proportion of those identified as high risk, genetic testing for cancer susceptibility (Campbell *et al*, 1995; Evans *et al*, 1994). Referrals to these services include not only those at high genetic risk, but also the much more frequent cases where risk is slightly increased or not significantly different from that of the general population. However, there is a lack of evidence regarding appropriate models of cancer genetics service provision for women who are at different levels of familial breast cancer risk.

Previous research illustrates the high level of interest in genetic testing among at-risk populations (Durfy *et al*, 1999; Jacobsen *et al*, 1997; Lerman *et al*, 1995). Although genetic testing is often regarded as the primary endpoint of cancer risk assessment services, lower risk individuals can gain useful information and support from these services without such testing (Kelly, 1999). Many women with a family history of breast cancer perceive themselves to be at high risk (Evans *et al*, 1993; Kash *et al*, 1992; Lerman *et al*, 1993) and may seek genetic advice in order to reduce anxiety about personal risk (Brain *et al*, 2000a). Indeed, perceptions of increased risk and associated anxiety may be more important than actual risk in motivating individuals to approach cancer genetics services (Lerman *et al*, 1994; Petersen *et al*, 1999). Therefore important aims of genetic risk assessment and counselling are to communicate accurate risk information and alleviate anxiety.

There are clear resource implications of providing widespread cancer genetics services and an urgent need for evidence regarding appropriate service provision for women at high risk and those at lower risk of breast cancer (Report by the expert advisory group on cancer to the Chief Medical Officers of England and Wales, 1995; Report of a working group on cancer genetics services, 1998). The present study addresses this issue by comparing the psychological impact of specialist genetic risk assessment with that of standard surgical provision on women identified as being at different levels of breast cancer risk. An earlier report of this randomized trial found few psychological effects of receiving specialist genetic assessment compared with surgical provision, with reductions in anxiety regardless of whether women received detailed genetic information (Brain *et al*, 2000b). In the current paper, we report secondary analyzes of these data in order to examine the impact of specialist genetic versus surgical assessment on primary outcomes (anxiety, worry and perceived risk) and secondary outcomes (interest in genetic testing and satisfaction) in subgroups of women stratified as low, moderate, or high risk. It was predicted that, regardless of their group allocation, anxiety and perceived risk would be higher in women found to be at high risk compared to those at lower risk. It was further predicted that high risk women would be less satisfied with their consultation and that interest in genetic testing would remain high in high risk women but would decrease in those at lower risk.

## MATERIALS AND METHODS

Full ethical approval was granted on the basis that, at the time the study was initiated, genetic risk assessment and possible presymptomatic testing were not available for families at increased risk of breast cancer in Wales. At that time, standard practice involved surgical-led management. A consecutive series of women identified by their general practitioner or local breast surgeon as having a family history of breast cancer were referred to the TRACE project (Trial of genetic assessment in breast cancer) during an 18-month period from 1996 to 1997. A family history of breast cancer was defined as a first-degree female relative diagnosed with breast cancer before age 50 years, a first-degree female relative with bilateral breast cancer at any age, two or more first-degree relatives with breast cancer, or a first- and second-degree relative with breast cancer. Women were excluded from the trial if they had a personal history of breast cancer, had previously received genetic counselling, or were not resident in Wales. Written informed consent was obtained from each participant. The consent form affirmed patient confidentiality and the right to withdraw from the research study at any time without affecting medical care. Further details of the referral procedure are reported in Brain *et al* (2000a).

Figure 1

**Figure 1:**
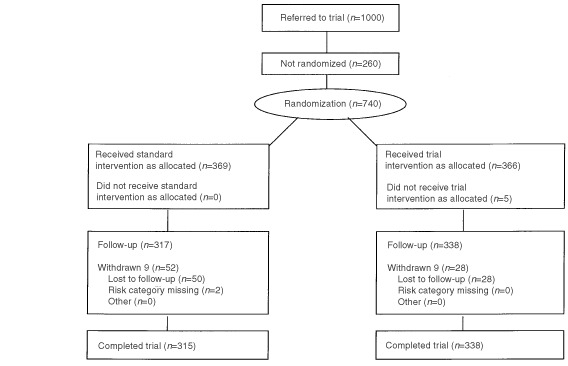
Trial profile.

illustrates the flow of patients through the trial. One thousand women were referred and sent a baseline questionnaire. Seven hundred and thirty-five randomly assigned participants in both study groups were given a questionnaire to complete after clinic, which was returned by 655 women. Factors associated with participation bias are reported in an earlier article (Brain *et al*, 2000b). The study was powered to detect a 0.5 difference at the 5% level between risk groups in primary outcomes.

At baseline, participants completed the state scale of the State-Trait Anxiety Inventory (Spielberger, 1983), the Breast Cancer Worry scale (Lerman *et al*, 1991a,b), and two scales on which they indicated their level of perceived risk and interest in obtaining genetic testing. The Breast Cancer Worry scale is a 6-item scale that assesses frequency of concerns about developing breast cancer and the impact of cancer worry on mood and daily functioning. It has high internal consistency (Cronbach's alpha=0.86). Higher worry scores have been associated with inappropriate adherence to mammography screening (Lerman *et al*, 1991b) and breast self-examination (Brain *et al*, 1999) in at-risk women. Two items derived from previous research (Champion, 1984; Fallowfield *et al*, 1990; Lerman *et al*, 1991a) were used to assess perceived risk (alpha=0.71). Interest in genetic testing was assessed using three categories (1=want a genetic test, 2=do not want a genetic test, 3=uncertain). Immediately after clinic, participants completed the same measures along with the 12-item Satisfaction with Genetic Counselling Questionnaire (Shiloh *et al*, 1990). This assesses three dimensions of patient satisfaction: (i) instrumental (the extent to which the patient believes the doctor has the required skills and gives the required treatment and reassurance) (alpha=0.69), (ii) affective (satisfaction with personal qualities of the doctor) (alpha=0.79), and (iii) procedural (satisfaction with administrative procedures such as waiting time) (alpha=0.65).

Individual randomization to the trial clinic or control clinic was based on a computer-generated sequence of random group assignments and took place on receiving the completed baseline questionnaire. In order to minimize response bias, there was no reference to group allocation in the baseline questionnaire or clinic appointment letter.

### Control group

Women randomized to the control clinic were seen by specialist surgical staff who included a breast surgeon and breast care nurse. A standard surgical protocol covered the following components: (i) breast cancer surveillance (clinical breast examination and, for women aged over 35 years, a mammogram) and advice on potential risk reducing strategies; (ii) surgical assessment of individual breast cancer risk; (iii) the option of entering the UK Tamoxifen Prevention Trial; and (iv) annual surgical follow-up involving breast cancer surveillance and advice. Referral for genetic counselling or presymptomatic genetic testing was not offered. Surgical assessment of risk was based on non-genetic information collected by the surgeon, including age, reproductive history and minimal family history. This information was presented to women by the breast surgeon in generalized terms and was stratified by the surgeon into one of three categories: low, moderate, or high risk.

### Trial group

Women randomized to the trial clinic were seen by a multidisciplinary team that combined clinical input from surgical staff (components 1, 3 and 4 of the control intervention) with specialist genetic risk assessment and counselling provided by a clinical geneticist and genetic nurse specialist. The surgical component of the trial clinic did not include a surgical assessment of risk. The genetic consultation involved genetic assessment of breast cancer risk, education and counselling about familial breast cancer, and the possible offer of presymptomatic testing for the BRCA1/2 gene. Assessment of risk was based on detailed family pedigree data that were collected and analyzed by the geneticist using the statistical model developed by Claus *et al* (1991). This provides a risk estimate based on the number of breast cancer cases in first and second degree relatives, the age at diagnosis of breast cancer in first and second degree relatives and the age of the presenting woman. The geneticist presented this information to women as a residual lifetime risk of breast cancer, expressed in percentage terms. Women were categorized by the geneticist as low risk (less than 10% residual lifetime risk), moderate risk (10–24%), or high risk (25% or more). Standardized UK guidelines for categorizing genetic risk were not available at the time this research was implemented. Chi-square analysis indicated group equivalence in the number of participants stratified as low, moderate, or high risk (see Table 1

**Table 1:**
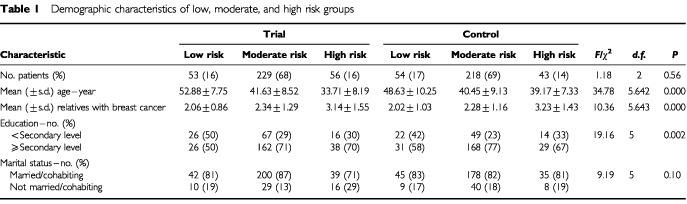
Demographic characteristics of low, moderate, and high risk groups

). Fifty women who were identified as high risk were offered mutation searching in an affected relative. A further six high risk women were not offered mutation searching because they did not have an affected relative available to give a blood sample. It was decided to retain this group in the statistical analysis since excluding them did not alter the pattern of findings. Full details of the clinical protocol are reported in Gray *et al* (2000).

### Statistical analysis

A previous report of this trial indicated baseline comparability of study groups (Brain *et al*, 2000b). Differences between risk groups in demographic characteristics were examined using one-way analysis of variance (ANOVA) or chi-square tests. A 2 (study group)×3 (risk group) repeated measures ANOVA was used to calculate the significance of group differences, changes over time, and possible interaction effects for the primary outcomes. Demographic characteristics in which risk groups differed were controlled and significant effects were followed up with paired *t*-tests or one-way ANOVA. The impact of study group and risk information on satisfaction (general factorial ANOVA) and interest in genetic testing (chi-square analysis) was examined. All results are based on 653 participants who received risk information and completed the questionnaires.

## RESULTS

### Sample characteristics

Table 1 displays descriptive statistics for the demographic characteristics of risk groups.

Risk groups differed significantly in education, age and family history of breast cancer.

### Impact of genetic risk assessment and counselling

Table 2

**Table 2:**
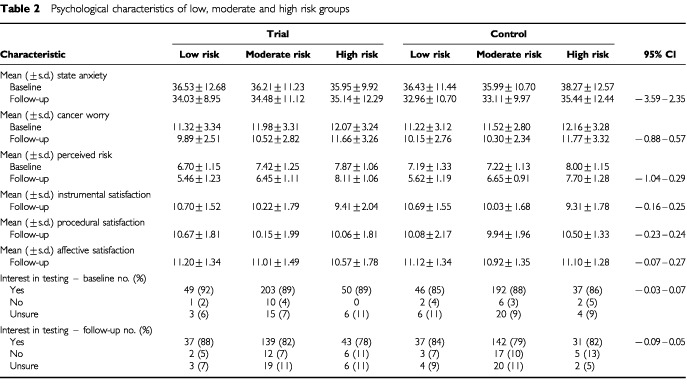
Psychological characteristics of low, moderate and high risk groups

displays descriptive statistics for psychological characteristics of risk groups at baseline and follow-up. Table 3

**Table 3:**
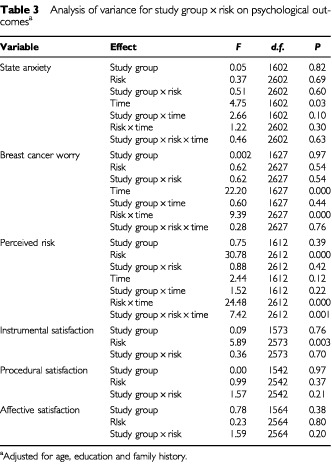
Analysis of variance for study group×risk on psychological outcomes^a^

shows the results of ANOVA.

#### State anxiety

There were no significant main or interaction effects of study group and risk information. There was a significant main effect of time, suggesting an overall decrease in anxiety from baseline to follow-up.

#### Breast cancer worry

There was a significant overall reduction in cancer worry from baseline to follow-up and a significant interaction between risk information and time. Post-hoc analysis indicated that cancer worry declined significantly in women at low risk (*t*(106)=5.92, *P*<0.001) and moderate risk (*t*(443)=12.13, *P*<0.001), but not in those at high risk (*t*(98)=1.67, *P*=0.10).

#### Perceived risk

There was a significant main effect of risk information. *Post-hoc* tests indicated that differences between risk groups were significant at baseline (*F*(2, 637)=17.48, *P*<0.001) and follow-up (*F*(2, 640)=128.56, *P*<0.001). At both assessments, perceived risk was significantly higher in the high risk group compared to the moderate or low risk groups, and was significantly higher in the moderate risk group compared to the low risk group. The main effect of risk information was modified by a significant risk×time interaction. While perceived risk declined significantly in women at low risk (*t*(101)=10.78, *P*<0.001) and moderate risk (*t*(431)=13.27, *P*<0.001), there was no significant change in perceived risk in those at high risk (*t*(96)=0.00, *P*=1.00). Post hoc analysis of the significant group×risk×time interaction effect indicated that within the trial group, perceived risk decreased significantly in women at low risk (*t*(49)=6.90, *P*<0.001) and moderate risk (*t*(221)=11.83, *P*<0.001). For high risk women in the trial group, there was a marginally significant trend towards increased perceived risk (*t*(53)=−1.72, *P*=0.09). Within the control group there was a significant decrease in perceived risk for women at low risk (*t*(51)=8.35, *P*<0.001) and moderate risk (*t*(209)=7.03, *P*<0.001), and a marginally significant trend towards reduced perceived risk in the high risk group (*t*(42)=1.83, *P*=0.07).

#### Satisfaction

There was a significant main effect of risk information, but not of study group, on instrumental satisfaction. Post-hoc analysis indicated that women at high risk reported significantly lower instrumental satisfaction than women at low or moderate risk, while those at moderate risk reported significantly lower satisfaction than those at low risk (*F*(2, 593)=13.80, *P*<0.001). There were no significant main or interaction effects on procedural and affective satisfaction.

#### Interest in genetic testing

Although there was a significant overall decline in interest (χ^2^=15.13, *P*=0.000, McNemar test), there was no significant difference between risk groups at baseline (χ^2^(10)=6.15, *P*=0.80) or follow-up (χ^2^(10)=4.93, *P*=0.90).

## DISCUSSION

An increasing number of women are seeking advice from health care professionals in connection with a family history of breast cancer. For a small subset of women, the family history strongly indicates the presence of an inherited gene mutation; for the majority of women who are at lower risk, the family history is not linked to specific inherited factors and genetic testing will not be of benefit. The present study provides evidence regarding appropriately structured service provision and effective use of genetic resources for women who are at different levels of familial breast cancer risk.

For most women who attend familial breast cancer clinics, beliefs about increased personal risk may be moderated by the provision of favourable risk information. The current findings suggest that in women at lower risk, anxiety and cancer-related concerns were reduced after receiving personal risk information and there were high levels of satisfaction, regardless of whether this information was based on a detailed genetic analysis. Although some studies have shown no effect of providing risk information on psychological outcomes (Kent *et al*, 2000; Lloyd *et al*, 1996), this may reflect differences in the way that risk information is communicated. Improved psychological outcomes may be a function of services that not only provide specific risk information, but also address patients' emotional needs by offering appropriate reassurance (Watson *et al*, 1998). Despite reduced anxiety and perceived risk, women at lower risk remained interested in obtaining genetic testing. Although lower risk individuals can gain useful information and support from cancer genetics services without having genetic testing, the desire for testing may be resistant to change and further exploration of this phenomenon is necessary.

For the minority of women who are at high risk, providing objective risk information may confirm beliefs about increased personal risk. The finding that general anxiety decreased in this group suggests that risk information, even when unfavourable, does not appear to generate clinically significant anxiety (Cull *et al*, 1999; Hopwood *et al*, 1998; Lerman *et al*, 1996). However, women at high risk remained concerned about breast cancer and were less satisfied with their consultation, regardless of study group. Hence unfavourable risk information may not lead to clinical anxiety, but persistent worries about breast cancer may compromise quality of life for women who already recognize that they are at increased genetic risk. It is possible that high risk women were kept in a state of uncertainty because genetic test results were not available to them at the time of this research. Although it was not within the scope of the current study to examine the impact of receiving genetic test results, prior research suggests that receiving test results may confer important psychological benefits by alleviating chronic worry and uncertainty about health in relation to breast cancer (Broadstock *et al*, 2000; Lerman *et al*, 1996, 1998). There was some evidence for an effect of study group on perceptions of personal risk in high risk women, suggesting that detailed genetic information and the offer of testing may generate increased concerns about risk. It was not possible to examine psychological impact separately for the small number of high risk women who could not be offered testing due to the absence of an affected relative available to give a blood sample for mutation searching. Further research is needed to explore psychological outcomes in high risk women who receive genetic test results compared with those who are awaiting results as well as those who cannot be offered testing.

It is acknowledged that the small effect of study group on perceived risk in high risk women may have reflected differences in methods of classifying and communicating risk that were used in the intervention and control arms of the trial. However, there did not appear to be an effect of different risk assignment methods on women at low or moderate risk, who were reassured regardless of study group. Further controlled studies are necessary to compare the reliability of different methods of assessing familial breast cancer risk.

The present findings have important resource implications. The multidisciplinary cancer genetics clinic at tertiary care level is a suitable environment for providing genetic information, counselling and testing to high risk families, but not all women with a family history need to be referred. Breast care specialists other than geneticists might provide initial assessments of risk for breast cancer, reassuring women at lower risk and targeting those at high risk for referral to specialist genetics clinics. In a report to the Department of Health on UK cancer genetic services (1998), recommendations were made for a filtered approach to service provision corresponding to the three levels of care described in the Calman-Hine report (1995). These are general practice (primary care), risk assessment at cancer unit level (secondary care), and specialist genetic services involving genetic counselling and, where appropriate, presymptomatic genetic testing at cancer centre level (tertiary care). This filtered approach is a vital first step in developing appropriate service structures and the selective use of specialist genetic resources in breast cancer and, increasingly, other common inherited cancers.

It is possible that the current findings reflect the high level of expertise of specialist staff who were involved in the project, which led to enhanced care in the control arm. It remains to be seen whether the findings will generalize to the community setting. Over the next few years, it is likely that initial genetic risk assessment will be conducted in different settings by primary care and, increasingly, nurse specialist staff. Further research must be undertaken to explore their potential role in the appropriate identification and management of at-risk women, the effectiveness of different strategies for assessing and communicating genetic risk information (Emery *et al*, 2000) and the acceptability of a filtered service structure to patients and providers. Recent consensus guidelines for assessing and stratifying familial breast cancer risk must be incorporated into clinical practice in order to minimize potential variation between the different health care professionals who are likely to be involved in the management of this patient group (British Association of Surgical Oncology Guidelines, 1998; Eccles *et al*, 2000). The education and training needs of health professionals must also be identified (Watson *et al*, 1999), since they may be required to not only communicate accurate genetic risk information but also address the psychological needs and concerns of individuals who approach them with concerns about a family history of cancer.
